# Bad Blood

**Published:** 1870-04

**Authors:** 


### BAD BLOOD.

DRAYMEN about breweries drink quarts, if not gallons, of beer every day, and by the time they are forty-five, the commonest scratch of a pin on the hand will not get well for months; if the skin is abraded or scraped off by a misstep or other accident, a running sore is sometimes established for the remainder of life; it is because the blood is bad; it is poor, too thick, and even poisonous.

Persons have poor blood when it is observed that scratches and cuts and bruises are a long time in healing, and this should be a friendly warning to correct that condition of things, because it shows there is but little vitality, little stamina, and disease of some kind is impending, especially of the typhoid type, and recovery will be slow, doubtful, and in many cases not possible.

The first step to be taken in all cases to get rid of bad blood, is to spend a large portion of daylight out of doors in remunerative labor or agreeable employment, or in journeying, on horseback being the best; this helps nature to work the bad blood out of the body, and at the same time gets up a good appetite and a vigorous digestion, which makes a pure blood to supply the place of the bad, and the man is well, without an atom of medicine or a dollar's expense.

This subject is pursued at length, and the diseases enumerated which are more particularly remedied by the application of the principle, in our last book, entitled HEALTH BY GOOD LIVING.

